# Development of a dynamic model for ventral hernia mesh repair

**DOI:** 10.1007/s00423-014-1239-x

**Published:** 2014-08-21

**Authors:** M. Siassi, A. Mahn, E. Baumann, M. Vollmer, G. Huber, M. Morlock, F. Kallinowski

**Affiliations:** 1 Department of General Surgery, Asklepios Klinik Harburg, Eißendorfer Pferdeweg 52, 21075 Hamburg, Germany; 2 Department of Biomechanics, Technische Universität Hamburg-Harburg, Schwarzenbergstrasse 95, 21073 Hamburg, Germany

**Keywords:** Laparoscopic ventral hernia repair, Dynamic simulation, Friction coefficient, Mesh fixation

## Abstract

**Introduction:**

The adequate way of mesh fixation in laparoscopic ventral hernia repair is still subject to debate. So far, simulation has only been carried out in a static way, thereby omitting dynamic effects of coughing or vomiting. We developed a dynamic model of the anterior abdominal wall.

**Materials and methods:**

An aluminium cylinder was equipped with a pressure controlled, fluid-filled plastic bag, simulating the abdominal viscera. A computer-controlled system allowed the control of influx and efflux, thus creating pressure peaks of up to 200 mmHg to simulate coughing and 290 mmHg to simulate vomiting. We tested fixation with tacks (Absorbatack, Covidien Deutschland, Neustadt a. D., Germany). The model was controlled for the friction coefficient of the tissue against the mesh and the physiologic elasticity of the abdominal wall surrogate.

**Results:**

The model was able to create pressure peaks equivalent to physiologic coughs or vomiting. Physiologic elasticity was thereby maintained. We could show that the friction coefficient is crucial to achieve a physiologic situation.

The meshes showed a tendency to dislocate with an increasing number of coughs (Fig. 4). Nevertheless, when applied in a plain manner, the meshes withstood more cough cycles than when applied with a bulge as in laparoscopic surgery.

**Conclusions:**

The dynamic movement of the abdominal wall, the friction between tissue and mesh and the way of mesh application are crucial factors that have to be controlled for in simulation of ventral abdominal hernia closure. We could demonstrate that patient specific factors such as the frequency of coughing as well as the application technique influence the long term stability of the mesh.

## Introduction

The occurrence of an incisional hernia is still a frequent complication of abdominal surgery, affecting up to 37 % of patients [1]. It is a widespread consensus, that hernia repair with mesh reinforcement of the abdominal wall is superior to suture techniques. Yet, the accurate way of mesh fixation, be it in the sublay or the intraperitoneal (IPOM) position, is still subject to debate [2]. Early postoperative mesh dislocation is considered a technical failure, since the static forces needed to disrupt a mesh fixation exceed those occurring physiologically in the abdomen when the fixation material is sufficiently selected. Extensive research has been carried out on the mechanical strength of different fixation techniques. Nevertheless, existing biomechanical models used industrial pressure testing devices [3]. These exert static pressure on the mesh fixation only. In these experiments, only very high pressures, exceeding fivefold the physiologic intraabdominal forces, lead to mesh or fixation disruption [4]. However, the movements of the abdominal wall are of a dynamic nature. During coughing, for example, there is a steep rise from a basal pressure of 1–10 mmHg of up to 200 mmHg with a subsequent, slower release. Vomiting exerts even higher pressures of up to 290 mmHg [5, 6]. These dynamic forces may exert forces on a fixed mesh that differ from those measured by static models. We therefore developed a model to simulate the dynamic forces of the anterior abdominal wall. Four factors were determined to be controlled for:

Dynamic pressure profile, simulating a physiologic cough

- Basal pressure in the abdominal cavity
- Elasticity of the abdominal wall model
- Friction between mesh and peritoneum

For the ease of use, a completely synthetic model was constructed and compared to a biological model using porcine abdominal wall preparations. In both models, we aimed at an elasticity of 25 % according to earlier studies on the elasticity of the abdominal wall [7]. We then tested a commercially available hernia mesh for dislocation in different way of application and fixation.

## Materials and methods

### Dynamic pressure profile and basal pressure

In order to exert controlled pressures of up to 290 mmHg, a hydrostatic system was designed. The abdomen was simulated by an aluminium cylinder of 15.8 × 26 cm inner diameter. The bottom plate was inserted in a floating manner. Underneath it, a force measurement capsule was inserted. The cover plate had a 16 × 16 cm frontal opening to allow for silicone plates or porcine abdominal wall preparations to be mounted. The abdominal viscera were simulated by a water-filled plastic bag of 9.5-l content and an estimated compliance of 0.1 l/kPa. Care was taken to completely evacuate all air during filling. This bag was connected to a water supply with a pressure-controlled valve system. Inflow and drainage were controlled by voltage-controlled valves. A computer-controlled voltage amplifier (PICA, Peekel Instruments, Rotterdam, Netherlands) was steered by specially developed software. The influx valve would open until a predefined maximum pressure was reached. After automatic closure of the influx valve, the output valve would open until the pressure decreased to 5 mmHg, thus simulating the basic intraperitoneal pressure. The resulting pressures were assessed by a force measurement capsule (Model 8524, 2 KN, Burster, Gernsbach, Germany) under the bottom plate of the model. Increases in pressure resulted in higher output voltages. The voltage was digitalized (I/O-card AT-MIO-16XE, National Instruments, Munich, Germany) and recorded with a self-programmed software under Lab View 7.1, National Instruments, as above). The conversion of the measured amplitudes into pressure and force values was calculated by the software.

### Measurement of friction coefficient

The friction coefficient was measured with a polypropylene mesh (TiMesh, pfm-medical, Köln, Germany) against porcine peritoneum in a dry state and with the use of different lubricants. The mesh was pressed against the peritoneum with a standardized weight (198 g) and then pulled with a handheld force measurement device (Model 325 TesT, Erkrath, Germany). The minimum force required to cause movement of the mesh was then recorded. The measurement was repeated five times consecutively, and the mean was calculated. The friction coefficient μ was calculated as μ = *F*_F_/*F*_N_, *F*_F_ being the friction force exerted on the mesh and *F*_N_ being the standardized normal force of 198 g pressing the mesh against the “peritoneum”. Since the peritoneal fluid is in equilibrium with the serum, we used bovine serum as a readily available reference for the different lubricants to define the minimally necessary lubrication [8].

### Elasticity of abdominal wall model

To simulate the anterior abdominal wall, first, a silicone pad as a synthetic model was used. In a second step, standardized porcine abdominal wall preparations (30 kg/1 year old pigs) were used. In order to simulate the physiological elasticity of the human abdominal wall, we demanded that the defects should not widen more than 25 % during coughing.

### Silicone model

The silicone pad could easily be mounted on the aluminium cylinder. A hole of 5-cm diameter was cut into the pad to simulate a hernia, using a custom made punch. Nevertheless, when exposed to physiologic pressures, the opening widened by more than 25 %. Therefore, it was reinforced with a synthetic rubber (Ethylen-Propylen-Dien-Monomer (EPDM) 90) plate as a skin surrogate. With this composite, the “hernia” widened by a maximum of 25 % when exposed to pressures of up to 290 mmHg. For friction testing, the inner layer was covered with porcine peritoneum (Fig. 3).

### Porcine model

In a second step, porcine abdominal wall preparations were used. We used specimens from 1 year old pigs of approximately 30 kg weight. To ensure the fixation in the mounting ring, the skin and the external rectus sheath were removed. The skin was replaced by an EDPM 90 pad. This composite layer could be tightly fixed in the mounting ring of the aluminium cylinder. The widening under pressure again did not exceed 25 %.

### Mesh application

The tests were run with a commercially available, titanium-coated polypropylene (TiMesh, pfm-medical, Köln, Germany. The mesh was cut in a way that an overlap of 5 cm to all sides was ensured. Distance markers were applied to the meshes in 1-cm steps to enable quantification of mesh migration. The meshes were applied plainly and with a standardized bulge using a 6-cm ball to simulate the abdominal distension when pneumoperitoneum is present during laparoscopy.

### Fixation techniques

The meshes were first applied without fixation in both models. In further tests, the meshes were fixed with eight staples (Absorbatack®, Covidien Deutschland, Neustadt a. D., Germany).

## Results

### Dynamic pressure profile

**Fig. 1:**
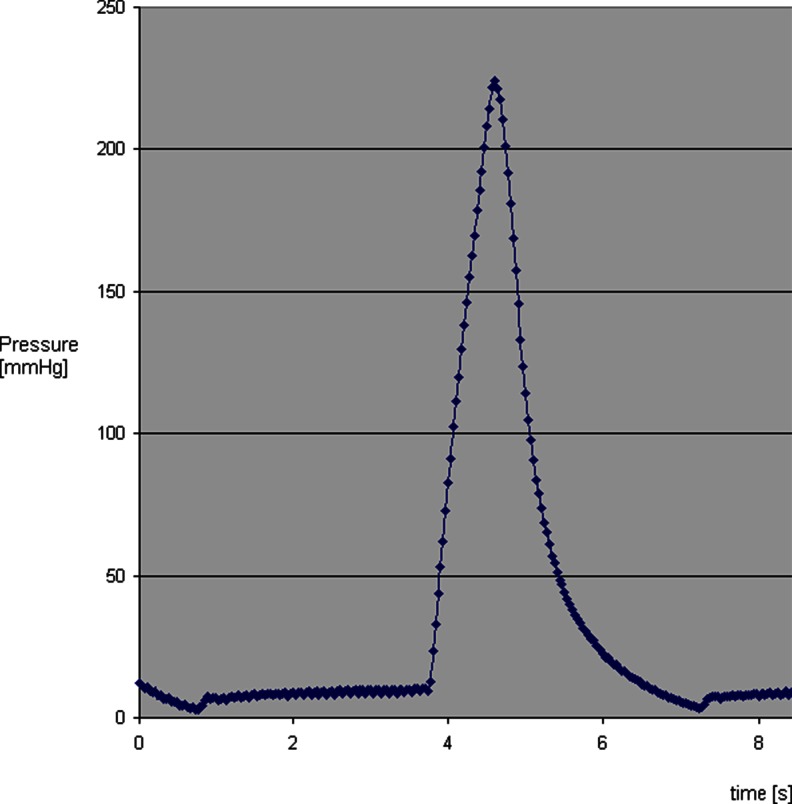
Pressure curve obtained by the model with no mesh inserted

The model was first run without a mesh inserted. After an inflow time of 833 ms, a peak pressure of 224 mmHg was reached. After a release time of 1,999 ms, the pressure resolved to the baseline level. The output valve closed at 5 mmHg, thus maintaining a physiologic basal pressure. The pressure curve is shown in Fig. 1.

### Friction coefficient

**Fig. 2:**
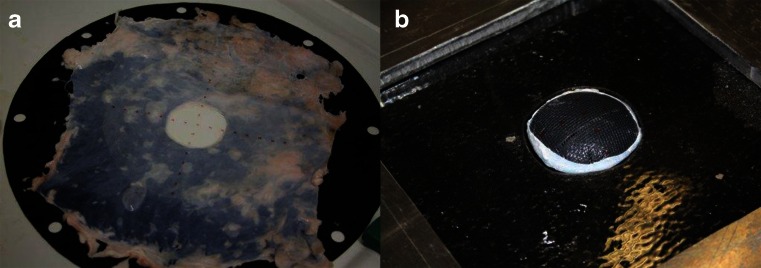
Inside view of the preparation of the silicone model with porcine peritoneum and mesh applied for friction testing. **b** Outside view of the silicone model with pressure applied, causing a bulge but no dislocation in the dry model with no additional lubricant applied

We measured the friction coefficients of polypropylene mesh against dry peritoneum and lubricated by mineral oil (Castrol EP 80 W, Deutsche Castrol, Hamburg, Germany), industrial machine fat, medical white vaseline, Margarine (Rela-Werke Fritz Busch GmbH, Ludwigsstadt, Germany), bovine serum, and mid-chain oil (Freka MCT Oil, Fresenius-Kabi, Bad Homburg, Germany) (Fig. 2). The friction coefficients are depicted in Table 1.

**Table 1:** Static friction coefficients of titanium-coated polypropylene mesh against porcine peritoneum with different lubricants

Lubricant	Friction coefficient [μ]
dry	0.81
Castrol EP 80 W oil	0.18
Industrial machine fat	0.08
Medical Vaseline	0.17
Margarine	0.04
Bovine serum	0.43
Freka MCT oil	0.03

All tested lubricants showed a lower friction coefficient than bovine serum. Yet, mineral oil, MCT Oil, and bovine serum were too liquid and thus could not be applied properly for the experiments. Therefore, medical vaseline was chosen as a standardized and easily applicable lubricant for the further tests.

### Silicone model

A polypropylene mesh was inserted into the silicone/EPDM composite with an overlap of 5 cm around the “hernia” edge without further fixation. In the dry model, there was no dislocation after 200 simulated coughs. After the application of lubricant, unfixed meshes dislocated after the first cough. We did not carry out fixation tests, because the staples could not be applied properly.

### Porcine model

**Fig. 3:**
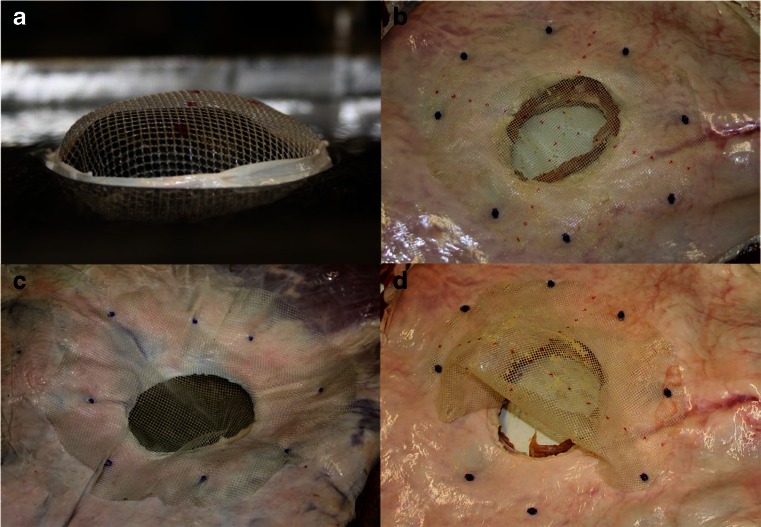
**a** Mesh fixed with eight tacks and a standardized bulge to simulate pneumoperitoneum. **b** Mesh applicated plainly. **c** Abdominal wall model with mesh in place after 300 simulated coughs. **d** Mesh dislocation due to tears in the mesh at the fixation points

**Fig. 4:**
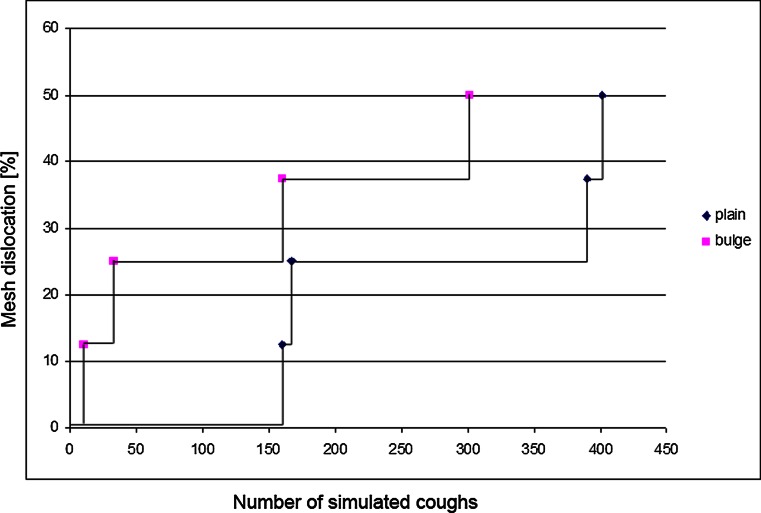
Percentage of meshes dislocating when applied either in a plain way or with a standardized bulge related to the number of coughs

Polypropylene meshes were applied in the same manner. Again, all unfixed meshes dislocated after the first simulated cough when the peritoneal surface was lubricated. In a pilot test, the meshes were fixed with four staples. With this fixation, all meshes dislocated (data not shown). The meshes were then fixed with eight staples. The tests were first performed running 300 cycles. When applied in a plain manner, two out of eight meshes dislocated. We then increased the number of cycles to 400. In this setting, four out of eight meshes dislocated after a median of 278.5 coughs. When applied with a bulge, four out of eight meshes dislocated after a median of 96.5 coughs (Figs. 3 and 4).

## Discussion

The movement of the anterior abdominal wall, that can lead to mesh migration after laparoscopic ventral hernia repair, is a dynamic process.

We demonstrated that beyond force, numerous factors affect the stability of the mesh application. The friction between mesh and peritoneum is a crucial factor. We showed that both synthetic and biologic models have an unphysiologically high friction coefficient. In our experiment, even unfixed meshes showed no migration when the material was not lubricated. The friction coefficient of dry peritoneum, as it is in all ex vivo experiments, is twice as high as after lubrication with serum, the most physiological surrogate for peritoneal fluid. Nevertheless, we could not use serum for the model, because of its low viscosity. For practical reasons, medical vaseline was chosen as a lubricant. Its friction coefficient is even lower than that of serum. In this way, experiments with our model cannot underestimate the quality of any given fixation system. We showed therefore, that a standardized and physiological lubrication is essential for the testing of mesh fixation. For this measurement, the silicone model proved an easy to use dry-lab simulation. We therefore recommend it for friction testing of different materials. Since the underlying EPDM sheet has a different structure than muscle, we did not consider it appropriate for the testing of staples. Nevertheless, the fixation of different meshes with glue, as discussed below, may also be done on this model.

We also controlled for the effect of abdominal distension caused by the application of a pneumoperitoneum during laparoscopy. It is usually suggested to lower the intraabdominal pressure as far as possible during mesh application, in order to achieve a plain position of the mesh. This hypothesis was supported by our tests. Nevertheless, we could demonstrate a curve of decreasing stability dependent on the number of coughs. If the mesh was applied in a plain manner, the dislocation occurred later. We could therefore demonstrate for the first time a “dose-dependent” correlation between abdominal wall movements and mesh dislocation. The results also show that patient factors play a major role. The plainly applied meshes dislocated only after a high number of coughs. Therefore, postoperative coughs should be reduced as far as possible. This has implications especially for the anesthesiological management as well as in patients with chronic pulmonary disease.

If these factors are taken into account, the mere mechanical stability is easily achieved. In our model, fixation with only eight staples ensured a safe fixation. This again demonstrates, that static force alone cannot explain the reasons for mesh migration. Anyway, the fixation with eight tacks is of course a minimum prerequisite. More staples may be necessary to prevent entrapment of small bowel, etc.

The use of glue for mesh fixation may avoid some of the disadvantages of tacks, such as adhesion formation and induction of pain [9]. The use of fibrin glue on intact peritoneum may not produce a safe fixation [10]. The use polyacrylate glue in our model showed a similar fixation quality compared to staples. Taking into account that static experiments suggest that the fixation strength depends on the combination of glue and mesh [11], we found evidence, that the combination of Ti-Mesh and Glubran yields satisfactory results even in a dynamic model.

The model has a few shortcomings. Firstly, the time, in which the pressure increases and decreases, is a lot longer than in a physiologic cough. This difference could not be eliminated due to technical reasons. Since the mass of the mesh and the tacks, is low, its mass inertia is also low, thereby minimizing velocity effects.

Another potential drawback is the different structure of porcine and human abdominal walls. In our specimens, we found the subperitoneal fat to be thicker than in humans. With the use of young pigs of standardized age and weight, we tried to control for this factor. Nevertheless, porcine abdominal wall samples can only be used as a surrogate for human abdominal wall. The alternative would be live animal experiments, which are too complex and costly for large scale experiments. Therefore, our model is an economic, yet physiologic means of hernia simulation. In addition, we discovered some widening of the experimental hernia when pressure was applied to the model. This is due to the fact, that then cadaveric material we used naturally lacks the muscle tone of an abdominal wall in vivo. There are no data on the behaviour of a hernia during coughing in vivo. We assumed that in spite of the muscular tone, there may be some widening. In our experiment, we limited the widening to 25 % in order to achieve reproducible results.

## Summary

In our model, for the first time, we simulated the complex dynamic processes exerted on meshes just after the completion of ventral abdominal hernia repair: coughing, ambient movements, jumping and others. We could demonstrate that static force measurements, as used in previous studies may yield misleading results. The dynamic nature of abdominal wall movements, the friction of the mesh against the peritoneum and the dynamic widening of the ventral hernia must be taken into account in order to obtain correct results in future experiments. In our preliminary study, the measurements showed that the mesh should be applied in a plain manner with no bulge. Nevertheless, regardless of the way of fixation, there is a “dose-dependent” tendency of the mesh to dislocate with an increasing number of coughs. This may carry practical implications for the perioperative therapy of patients with ventral hernia. The dynamics of pressure changes, the friction coefficient of the materials and the elasticity of the abdominal wall exert important influences of the safety of the repair used. Further experiments on other meshes and fixations devices are necessary in order to develop clear guidelines for securely anchoring mesh materials under different conditions.
